# Incidences of community onset severe sepsis, Sepsis-3 sepsis, and bacteremia in Sweden – A prospective population-based study

**DOI:** 10.1371/journal.pone.0225700

**Published:** 2019-12-05

**Authors:** Lars Ljungström, Rune Andersson, Gunnar Jacobsson

**Affiliations:** 1 Department of Infectious Diseases, Institute of Biomedicine, Sahlgrenska Academy, Gothenburg University, Gothenburg, Sweden; 2 Region Västra Götaland, Skaraborg Hospital, Department of Infectious Diseases, Skövde, Sweden; 3 CARe–Center for Antibiotic Resistance Research, Gothenburg University, Gothenburg, Sweden; University of Pittsburgh, UNITED STATES

## Abstract

**Background:**

Sepsis is a major healthcare challenge globally. However, epidemiologic data based on population studies are scarce.

**Methods:**

During a 9-month prospective, population-based study, the Swedish Sepsis-2 criteria were used to investigate the incidence of community onset severe sepsis in adults aged ≥18 years (N = 2,196; mean age, 69; range, 18–102 years). All the patients who were admitted to the hospital and started on intravenous antibiotic treatment within 48 hours were evaluated. Retrospectively the incidence of sepsis according to Sepsis-3 criteria was calculated on this cohort.

**Results:**

The annual incidence of community onset severe sepsis in adults at first admission was 276/100,000 (95% CI, 254–300). The incidence increased more than 40-fold between the youngest and the oldest age group, and was higher for men than for women. The respiratory tract was the most common site of infection (41% of cases). Using the Sepsis-3 criteria, the annual incidence of sepsis was 838/100,000 (95% CI, 798–877), which is 3-fold higher than that of severe sepsis. The main reason for the discrepancy in incidences is the more generous criteria for respiratory dysfunction used in Sepsis-3. Bacteremia was seen in 13% of all the admitted patients, giving an incidence of 203/100,000/year (95%, CI 184–223), which is among the highest incidences reported.

**Conclusions:**

We found a high incidence of community onset severe sepsis, albeit lower than that seen in previous Scandinavian studies. The incidence increased markedly with age of the patient. The incidence of community onset sepsis according to the Sepsis-3 definition is the highest reported to date. It is 3-fold higher than that for severe sepsis, due to more generous criteria for respiratory dysfunction. A very high incidence of bacteremia was noted, partly explained by the high frequency of blood cultures.

## Introduction

Sepsis is a common healthcare problem. Worldwide, as many as 33 million people are affected annually, and case fatality rates are high [1]. Due to methodologic challenges, data on the incidence of sepsis have been difficult to compile. Owing to differences in sepsis criteria and methodologies used, as well as in the populations studied, the incidences reported have varied by a factor of more than one hundred [2]. While chart-based prospective population studies are considered Gold standard [3], they are often difficult to perform, especially on large populations. A prospective chart-based study on medical emergency patients in Denmark reported an incidence of community-acquired severe sepsis of 457/100,000 (95% CI, 430–485) person-years at risk [4]. From the Faroe Islands, a self-governing country of the kingdom of Denmark, a similar study reported an incidence of community acquired severe sepsis of 644/100,000 (95% CI, 623–668) person-years at risk [5]. A retrospective, modified, chart-based population study carried out in Sweden, which examined four evenly distributed days during one year, found an incidence of all severe sepsis of 687/100,000 (95% CI, 549–824) [6]. According to the same study, the incidence of sepsis according to the Sepsis-3 definition was 780/100,000 (95% CI, 633–926).

In 2016, a new international consensus definition of sepsis, commonly termed ‘Sepsis-3’, was launched. Sepsis is now defined as “life-threatening organ dysfunction caused by a dysregulated host response to infection” [7]. Thus, “organ dysfunction” is incorporated into the very definition of sepsis, making the term ‘severe sepsis’ no longer relevant. The criterion for organ dysfunction is a ≥2 point increase from patient baseline in the Sequential (sepsis-related) Organ Failure Assessment (SOFA) score [8], corresponding to a case fatality rate (CFR) of ≥10%. In addition to the new Sepsis-3 definition, a new definition and new criteria for septic shock were published. Adult septic shock is now defined as a subset of sepsis “and can be identified using the clinical criteria of hypotension requiring use of vasopressors to maintain mean blood pressure of 65 mmHg or greater and having a serum lactate level greater than 2 mmol/L persisting after adequate fluid resuscitation” [9].

The aim of this study was to estimate the incidences of community onset severe sepsis and of bacteremia by performing a prospective, consecutive, chart-based population study in a well-defined area in southwestern Sweden. Retrospectively we used this cohort to estimate the incidence of sepsis according to the new Sepsis-3 criteria.

## Patients and methods

The study was approved by the Ethical Review Board at the University of Gothenburg (permit 376/2011). Due to the observational nature of the study, no individual written informed consent was needed for cultures and biochemistry analyses that were included in the routine patient care. No individual consent was needed for reviewing the patients’ electronic health record for the purpose of this study.

Skaraborg Hospital is a 640-bed secondary-care public hospital in southwestern Sweden and is the only hospital serving 256,700 inhabitants, of which 206,900 are adult ≥18 years of age (Dec 31, 2011). The age and gender distributions of the study population are listed in S1 Table.

The hospital sees approximately 60,000 annual visits to its Emergency Department (ED) and has approximately 24,000 admissions. An electronic health record is used throughout the hospital. One accredited private laboratory serves the hospital with diagnostics related to both clinical chemistry and clinical microbiology. The Emergency Medical Service (EMS) is public and is the same throughout the catchment area. The EMS and ED use the same triage system, the Medical (Rapid) Emergency Triage and Treatment System (METTS/RETTS) [10], which includes documentation of vital signs. These sources were used to retrieve information to assess the presence or development of severe sepsis or septic shock.

During a 9-month period, from September 8, 2011 to June 7, 2012, all adult permanent residents admitted to the hospital and starting on intravenous antibiotic treatment within 48 hours, based on clinical suspicion of bacterial infection, were evaluated for the presence or development of community onset severe sepsis or septic shock during the first 48 hours. All admissions were evaluated, but the incidences were calculated using the first episode for each patient only.

Severe sepsis or septic shock was diagnosed using the 2011 Swedish definitions of severe sepsis, and septic shock [11] (S2 Table). Notably, for the diagnosis of severe sepsis, a verified infection was sufficient, even in the absence of 2/4 Systemic Inflammatory Response Syndrome (SIRS) criteria [12]. The Swedish definition states that status changes should have occurred from the starting point of fairly normal organ function and should not be expected to have any cause other than the systemic inflammatory response.

For the diagnosis of SIRS, the values at baseline were used. The baseline was defined as the highest pulse rate, the highest respiratory rate, the highest or lowest leukocyte count, and the highest or lowest temperature documented by the EMS, in the ED or in the ward before the initiation of intravenous antibiotic therapy.

Retrospectively, this cohort was evaluated for sepsis according to the Sepsis-3 criteria (S3 Table). To calculate the SOFA score for each patient, the values that showed the greatest deviation during the first 48 hours after admission were used. The SOFA score for respiration for each patient was corrected for the level of supplementary oxygen. All patients with ≥2 points in the SOFA score were corrected for baseline values, when available. Otherwise face values were used.

The baseline value for any particular biomarker was defined as the most deviant value documented by the EMS, in the ED or in the ward, at any time before the start of intravenous antibiotic treatment.

### Comorbidities

Comorbidities were divided into eight categories, all of which had to have been previously diagnosed: 1) Chronic cardiovascular disease, 2) chronic respiratory disease, 3) chronic renal disease, 4) chronic liver disease, 5) diabetes mellitus, 6) malignancy, ongoing or within the past 5 years; 7) immunosuppression, and 8) Other comorbidities (S4 Table).

### Inclusion and evaluation

For each patient considered for inclusion, oral and written information about the study was provided to the patient or a close relative by the receiving nurse. The nurse initiated a protocol that was used throughout the hospital stay. All the protocols were evaluated by one of the two senior specialists in infectious diseases, LL or GJ, both of whom have >15 years of clinical experience.

### Blood cultures and biochemistry

In line with the hospital’s policy, blood cultures were drawn from each patient before the initiation of intravenous antibiotic treatment. For patients with sepsis from an unknown focus, samples from the urine and the respiratory tract were cultured whenever possible. Other samples were collected at the discretion of the treating physician. Microbiologic culturing was performed as previously described [13]. For the detection of Influenza A virus, Influenza B virus, Human orthopneumovirus (previously named Human respiratory syncytial virus), and for *Mycoplasma pneumoniae*, laboratory-developed polymerase chain reaction (PCR) techniques were used throughout the study period. During 10 weeks of the winter season, two different respiratory Multiplex PCR assays for respiratory pathogens were also used, as previously described [14]. Routine biochemistry analyses included a full hemogram, plasma electrolytes, creatinine, albumin, bilirubin, liver enzymes, and C-reactive protein (CRP). These were measured with the ADVIA 1800/2400 Clinical Chemistry System (Siemens Healthcare GmbH, Erlangen, Germany), and as previously described [15]. Additional routine biochemistry analysis included measurement of the erythrocyte sedimentation rate, the neutrophil-to-lymphocyte count ratio (NLCR), prothrombin complex, and venous blood gas with lactate.

### Infection diagnosis

Community onset infection was defined as an infection diagnosed within 48 hours of admission, including both community acquired infections and healthcare-associated infections. A healthcare-associated infection was defined as an infection occurring in a patient undergoing hemodialysis or chemotherapy for a malignant disease within the past 30 days, or with an indwelling catheter or central line. The exclusion criteria were: previous admission within the preceding 7 days; an infection that was diagnosed >48 hours after admission to the hospital; not resident in the hospital area; any re-admission within 30 days for the same infection, and post-operative infections within 30 days of surgery. Definitions of the infection diagnoses are shown in S1 text. When available, definitions described in Swedish national guidelines were used [16].

### Subject dropout analysis

Two methods were used to approximate the proportion of patients that had been missed to be included during the study period. First, the laboratory provided a list of all the blood cultures performed during the study. Secondly, from the electronic health records we identified all patients who, during the month of March 2012, had received intravenous antibiotics within 48 hours of admission. These data were compared with the patients included in the study. Those identified that had not been included were evaluated in the study, provided that they could be assigned to the intended study population.

### Statistical analyses

Descriptive statistics are presented as means with standard deviations for continuous data and frequencies and percentages for categorical data. For ordinal data or skewed distributed data, the medians with quartiles are presented. Both parametric (*t*-test) and non-parametric (Mann-Whitney *U*-test) tests were used for univariate comparisons, depending on the types of data. Two-sided testing was performed for all the analyses, and a *p*-value <0.05 was regarded as statistically significant. The data were analyzed using the IBM SPSS ver. 22.0 software (SPSS Inc., Chicago, IL, USA).

## Results

### Patient selection and characteristics

**Fig 1:**
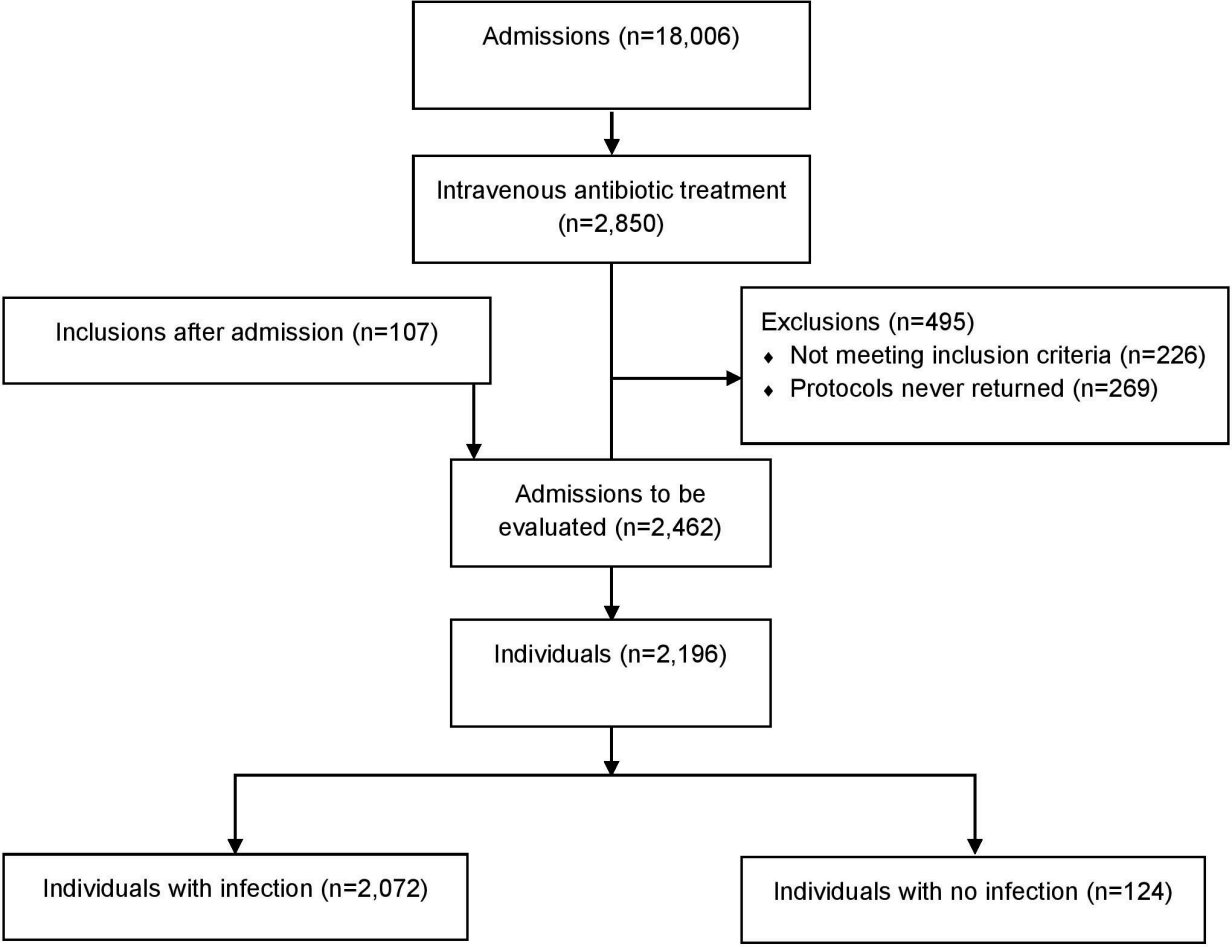
Patient selection flowchart for the present study.

Out of 18,006 admissions during the study period, 2,850 (16%) began intravenous antibiotic treatment. Exclusion criteria applied to 226 of the admissions, leaving 2,624 for evaluation. A dropout analysis for the month of March, using data from the electronic health records, identified 23 out of 343 (7%) admitted patients who had been missed for inclusion. The detection of positive blood cultures in the laboratory over the whole study period led to identification of 30 out of 315 (10%) who had been missed for inclusion. Another 54 patients were reported after admission. Thus, 117 admissions (4%) were identified that had initially been missed to be included, but were evaluated since they belonged to the intended study population. Overall, 269 patient protocols were never returned (9.4%). In all, 2,462 admissions remained for evaluation, representing 2,196 unique patients, of whom 213 (10%) had multiple entries (Fig 1).

Patient characteristics are shown in Table 1. The median age of patients with severe sepsis as well as Sepsis-3 sepsis was 78 years, which was significantly higher than for those who did not have severe sepsis, 72 years, or who did not have Sepsis-3 sepsis, 65 years. In all four groups, 53%–55% of the participants were men. In total, 124 patients did not have an infection and were excluded for incidence rates, but included in the analyses of vital signs and biomarkers at baseline, when sepsis was first suspected.

**Table 1:** Characteristics of the 2,196 consecutive patients admitted with clinical suspicion of bacterial infection and receiving intravenous antibiotic treatment within 48 hours of admission. Results for all patients and for those with severe sepsis and Sepsis-3 sepsis respectively.

Characteristics	Clinically suspected bacterial infection N (%)	Severe Sepsis N (%)	Not severe sepsis N (%)	*p-*value	Sepsis-3 sepsis N (%)	Not Sepsis-3 sepsis N (%)	*p*-value
**Individuals**	2,196	429 (20)	1,767 (80)		1,299 (59)	897 (41)	
**Age, years, mean (SD)**	69 (18)	74 (15)	67 (19)	<0.001	74 (15)	61 (20)	<0.001
**Age, years, median (IQR)**	73 (60–83)	78 (68–85)	72 (57–81)	<0.001	78 (67–85)	65 (47–77)	<0.001
**Males**	1,199 (55)	298 (53)	801 (55)	ns	708 (55)	491 (54)	ns
**No co–morbidities**	359 (16)	37 (9)	322 (18)	<0.001	119 (9)	240 (27)	<0.001
**Chronic cardiovascular disease**	1,183 (54)	274 (64)	909 (51)	<0.001	824 (63)	359 (40)	<0.001
**Chronic lung disease**	352 (16)	75 (18)	277 (16)	ns	263 (20)	89 (10)	<0.001
**Chronic kidney disease**	153 (7)	31 (7)	122 (7)	ns	87 (7)	62 (7)	ns
**Chronic liver disease**	13 (.6)	2 (.5)	11 (.6)	ns	8 (1)	5 (1)	ns
**Diabetes mellitus**	375 (17)	105 (25)	270 (15)	<0.001	257 (20)	118 (13)	<0.001
**Malignancy <5 years**	377 (17)	80 (19)	297 (17)	ns	241 (19)	136 (15)	0.002
**Immunosuppression**	106 (5)	20 (5)	86 (5)	ns	55 (4)	51 (6)	ns
**Other**	706 (32)	162 (38)	544 (31)	0.012	432 (33)	274 (30)	ns
**Patients with multiple admissions N (column %)**							
**1 admission**	1,982 (90)	392 (84)	1,590 (89)		1,154 (89)	828 (92)	
**2 admissions**	169 (8)	29 (13)	140 (8)		116 (9)	53 (6)	
**3 admissions**	39 (2)	7 (3)	32 (2)		25 (2)	14 (2)	
**≥4 admissions**	6 (.5)	1 (.6)	5 (.2)		4 (.4)	2	

SD, Standard Deviation; IQR, Interquartile range; SOFA, Sequential Organ Failure Assessment (score); ns, not significant.

### Incidence

**Fig 2:**
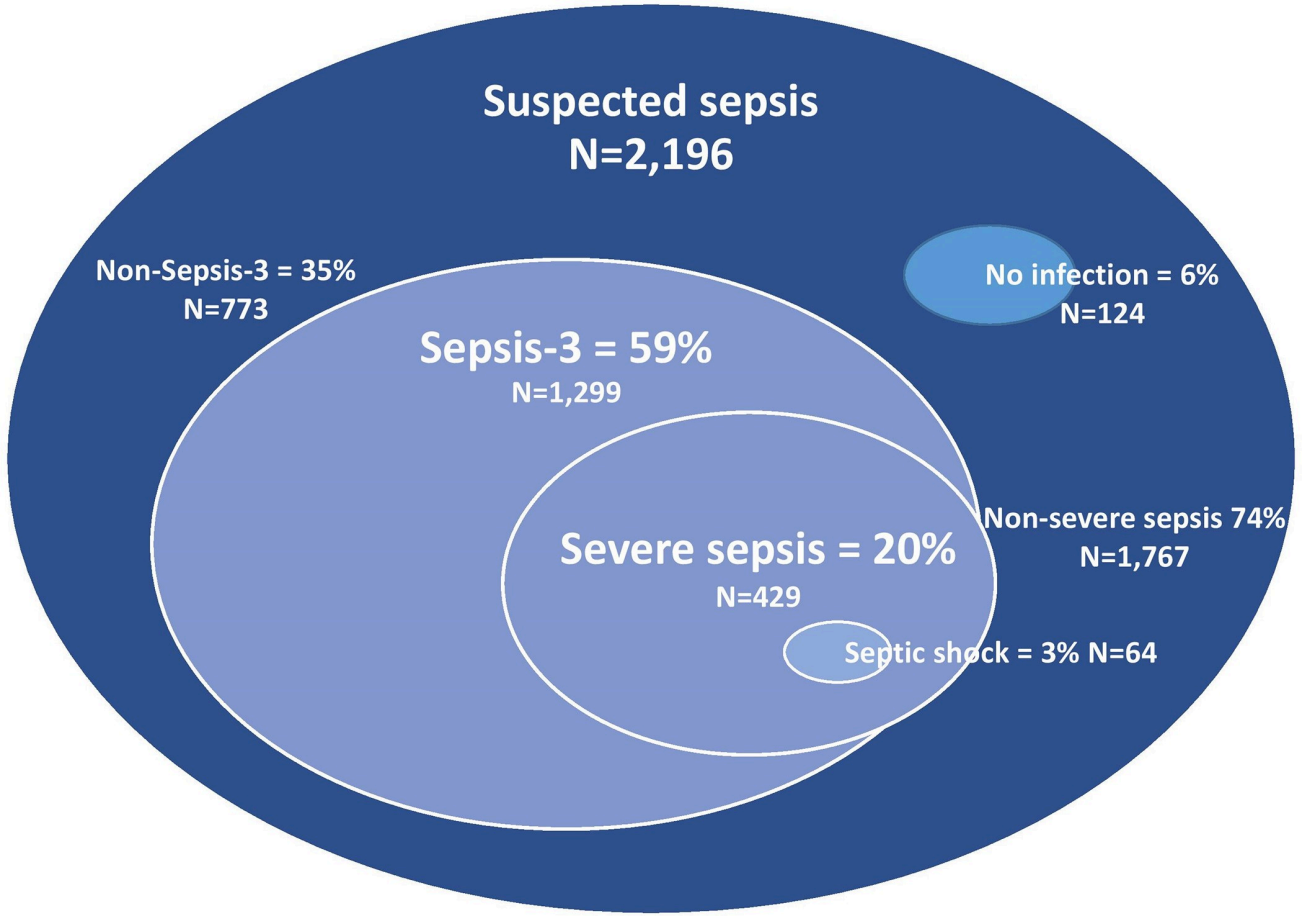
Distribution of severe sepsis and Sepsis-3 sepsis in a cohort of 2,196 consecutive patients with suspected community onset bacterial infection receiving intravenous antibiotic treatment.

At first admission, 429/2,196 patients (20%) fulfilled at least one of the Swedish criteria for severe sepsis within 48 hours, which is equivalent to an annual incidence of 276/100,000 (95% CI, 254–300). Sepsis as defined by Sepsis-3 criteria was identified in 1,299/2,196 patients (59%), equivalent to an annual incidence of 838/100,000 (95% CI, 798–877) (Fig 2).

**Fig 3:**
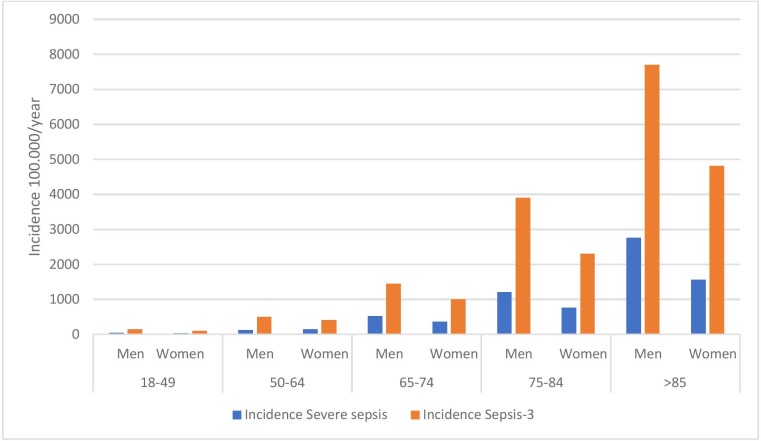
Incidence per 100,000 persons/year according to age group and gender of patients with severe sepsis (N = 429) and Sepsis-3 sepsis (N = 1,299).

The incidences of both severe sepsis and Sepsis-3 sepsis increased more than 40-fold from the youngest to the oldest age groups, and were higher for men than for women. The incidence of severe sepsis in men compared to women was: 46% higher in the 65–74 year age group; 59% higher in the 75–84 year age group; and 76% higher in the >85 year age group (Fig 3 and S1 Table). Of the 429 patients with severe sepsis, 393 (92%) also fulfilled the criteria for Sepsis-3.

Intravenous antibiotic treatment was started in the ED in 85% of the cases and in the general wards in 15% of the patients included. Healthcare-associated infections were identified in 10% of the patients.

### Comorbidities

The patients with severe sepsis were significantly more likely to suffer from cardiovascular disease, diabetes mellitus, and other comorbidities than those patients who did not have severe sepsis. The average number of comorbidities per age group increased from 0.6 in the 18–49 year age group to 1.7–1.9 in the older age groups.

### Vital signs

The baseline vital signs at first admission are displayed in Table 2. Hypotension was found in 179 patients, but on arrival only in 98. The remaining 81 patients developed hypotension within the first 48 hours. Of the 242 patients with cerebral dysfunction, based on a Reaction Level Scale (RLS) value of ≥2, 135 (56%) were classified as suffering from severe sepsis. Of the remaining 107 (44%) patients, 92 had an infection but were not classified as severe sepsis because of severe underlying cerebral disease, such as advanced Alzheimer’s dementia, multiple sclerosis, cerebrovascular disease, or brain tumor. Neither did they fulfill any other criteria for severe sepsis.

**Table 2:** Vital signs^1^ at baseline in 2,196 consecutive adult patients with suspected community onset bacterial infection treated with intravenous antibiotics. Results are presented also for patients with severe sepsis and Sepsis-3 sepsis. Values listed are medians (IQR) for all but RLS where the number of patients with RLS ≥2 are used.

	Suspected bacterial infection, all	Severe sepsis	Not severe sepsis	*p-*value	Sepsis-3 sepsis	Not Sepsis-3 sepsis	*p-*value
**Patients, N**	2,196	429	1,767		1,299	897	
**Heart rate (beats/min)**	99 (85–111)	107 (93–122)	97 (84–109)	<0.001	100 (88–115)	96 (83–107)	<0.001
**Systolic blood pressure (mmHg)**	130 (112–148)	116 (95–140)	133 (118–150)	<0.001	126 (108–145)	136 (120–150)	<0.001
**Respiratory rate (breaths/min)**	24 (20–30)	30 (24–36)	24 (20–28)	<0.001	26 (22–32)	20 (18–24)	<0.001
**Saturation (%)**	94 (90–96)	89 (83–94)	94 (91–96)	<0.001	91 (87–94)	96 (95–97)	<0.001
**Altered mental status (RLS** **≥2)**	242 *(11)*	135 *(30)*	107 *(6)*	<0.001	212 *(16)*	22 *(2)*	<0.001
**Temperature (°C)**	38.0 (37.1–38.8)	37.9 (37.0–38.8)	38.1 (37.1–38.8)	<0.01	38.1 (37.2–38.8)	37.9 (37.0–38.6)	<0.01
**History of fever**							
**Yes N (%)**	1,486 *(68)*	242 *(56)*	1,244 *(70)*		848 *(65)*	638 *(71)*	
**No N (%)**	448 *(20)*	99 *(23)*	349 *(20)*	<0.001	266 *(21)*	182 *(20)*	<0.001
**No information N (%)**	262 *(12)*	88 *(20)*	174 *(10)*		185 *(14)*	77 *(8)*	

^1^For any single vital sign, the percentage for which data were missing was <4%. IQR, Interquartile range; RLS, Reaction Level Scale.

### Laboratory results

The clinical chemistry results at first admission for 2,196 consecutive patients with suspected community onset bacterial infection treated with intravenous antibiotics are displayed in Table 3. For patients in general wards, there was rarely more than one daily assessment of biochemical markers, and for several markers there was only the initial assessment in the ED.

**Table 3:** Laboratory results at baseline for 2,196 consecutive adult patients admitted with suspected community onset bacterial infection. Results are presented also for patients with severe sepsis and Sepsis-3 sepsis. Values listed are medians (IQR)^1^.

	Suspected community onset bacterial infection	Severe sepsis	Not severe sepsis	*p-*value	Sepsis-3 sepsis	Not sepsis-3 sepsis	*p-*value
**Patients (N)**	2,196	429	1,767		1,299	897	
**Hemoglobin (g/L)**	130 (117–143)	129 (117–144)	131 (118–142)	ns	130 (117–142)	132 (119–143)	<0.001
**Leukocytes (× 10**^**9**^**/L)**	11.8 (8.6–15.7)	12.9 (9.3–17.7)	11.5 (8.5–15.1)	<0.001	12.4 (8.9–16.3)	11.3 (8.2–14.4)	<0.001
**Neutrophils (× 10**^**9**^**/L)**	9.3 (6.4–13.1)	11.5 (7.1–16.0)	9.0 (6.3–12.4)	<0.001	9.9 (6.8–13.7)	8.5 (5.9–11.9)	<0.001
**Lymphocytes (× 10**^**9**^**/L)**	1.0 (0.6–1.4)	0.7 (0.4–1.2)	1.0 (0.7–1.5)	<0.001	0.9 (0.5–1.3)	1.1 (0.7–1.6)	<0.001
**NLCR**	9.5 (5.3–16.8)	15.5 (8.2–26.3)	8.7 (5.0–15.1)	<0.001	11.1 (6.6–19.5)	7.3 (4.4–13.2)	<0.001
**CRP (mg/L)**	104 (40–177)	107 (40–213)	101 (40–103)	<0.032	109 (43–191)	96 (35–160)	<0.005
**Creatinine (μmol/L)**	81 (64–107)	100 (74–140)	78 (63–100)	<0.001	88 (69–123)	74 (60–89)	<0.001
**Bilirubin (μmol/L)**	11 (7–16)	12 (7–17)	10 (7–16)	<0.075	12 (8–18)	10 (7–14)	<0.001
**Platelets (× 10**^**9**^**/L)**	238 (184–303)	234 (173–304)	239 (186–302)	ns	228 (172–294)	250 (20–318)	<0.001
**INR**	1.1 (1.0–1.2)	1.1 (1.0–1.3)	1.1 (1.0–1.2)	<0.001	1.1 (1.0–1.3)	1.1 (1.0–1.2)	<0.001
**Lactate (mmol/L)**	1.8 (1.3–2.4)	3.2 (2.0–4.2)	1.6 (1.2–2.1)	<0.001	1.9 (1.4–2.6)	1.5 (1.2–2.1)	<0.001

^1^At baseline, the percentage for which data were missing was ≤7% for any parameter except INR, for which it was 9%; NLCR, Neutrophil-to-lymphocyte counts ratio; CRP, C-reactive protein; IQR, Interquartile range; INR, International normalized ratio; ns, not significant.

### SIRS criteria

At first admission, ≥2 SIRS criteria were present in 1,581/2,072 (76%) of the patients with an infection. Among the 429 patients with severe sepsis, 380 (89%) had ≥2 SIRS criteria.

### Organ dysfunction

**Fig 4:**
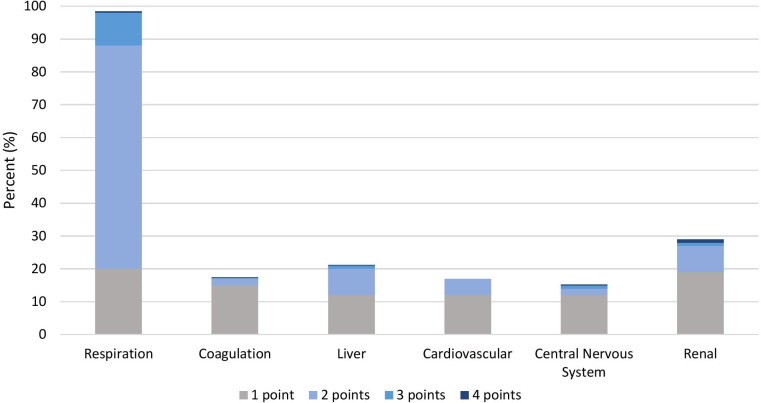
Distribution of points in the SOFA-score per organ system in 1,299 consecutive patients with Sepsis-3. The SOFA scores are corrected for the baseline levels.

The distribution of organ dysfunctions in severe sepsis and in Sepsis-3 sepsis, after correction for baseline values, are displayed in Table 4. Notably, the respiratory criterion for lung dysfunction was fulfilled in 160/429 (38%) patients with severe sepsis and in 1,011/1,299 (78%) patients with Sepsis-3 sepsis. The distribution of SOFA points during the first 48 hours after admission is shown in Fig 4. Out of the 1,299 patients with ≥2 points in the SOFA score, 173 (13%) patients accumulated two or more SOFA points through the addition of one point only for any organ system.

**Table 4:** Occurrence of organ dysfunction at first admission in 2,072 consecutive adult patients with community onset infection.

Organ dysfunction within 48 hours N (%)	Severe sepsis N = 429	Only organ dysfunction	Sepsis-3 sepsis N = 1,299	Only organ dysfunction
**Hypotension**	179 (42)	43 (10)	42* (3)	2 (.2)
**Hypoperfusion**	223 (52)	89 (21)	na	na
**Respiratory dysfunction**	160 (38)	39 (9)	1,011 (78)	805 (62)
**Renal dysfunction**	94 (24)	5 (1)	132 (10)	40 (3)
**Coagulation dysfunction**	47 (11)	7 (2)	26 (2)	58 (2)
**Altered mental status**	135 (31)	27 (6)	44 (3)	5 (.4)
**Hepatic**	8 (2)	0	121 (9)	6 (.5)
**Patients with number of organ dysfunctions:**				
**1**	210 (49)		916 (70)	
**2**	111 (26)		168 (13)	
**3**	58 (13)		34 (3)	
**>4**	50 (12)		10 (1)	

*Forty-two of 64 patients were treated in the ICU due to sepsis and hypotension, and were presumed to have received vasopressor treatment; na, not applicable.

**Fig 5:**
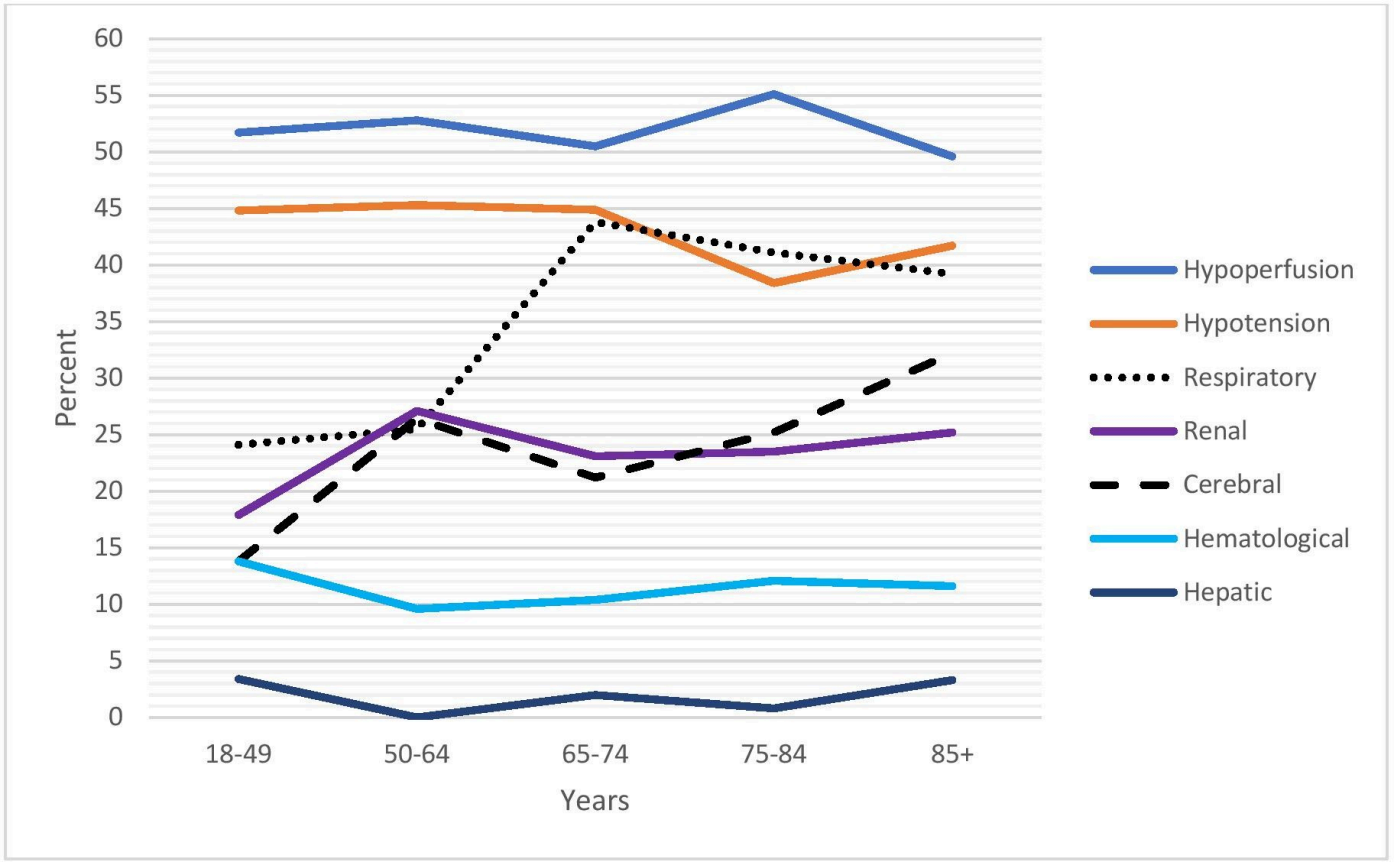
Organ dysfunction rates in 482 episodes with severe sepsis, classified according to age group.

In 429 patients with severe sepsis, the prevalence of organ dysfunction varied according to age group. Respiratory dysfunction and cerebral dysfunction increased with advanced age, but the remaining dysfunctions occurred at a constant rate regardless of age group (Fig 5).

### Microbiologic diagnoses

Using culturing or PCR of samples from the suspected focus of infection, a microbiological etiology could be defined for 944/2,072 (46%) patients with infection. Forty-six of the 944 (5%) had respiratory virus infection only, identified by multiplex PCR only. In patients with severe sepsis, an etiology could be identified in 50%. A bacterial etiology could be identified in 47% (203/429), more than half of these in the blood cultures, 62% (125/203). Influenza A virus was estimated to be the sole cause of the infection in 2% (10/429). In patients with Sepsis-3 sepsis, an etiology could be identified in 47%.

### Blood cultures

Blood cultures were obtained before the initiation of intravenous antibiotic treatment in 2,443/2,462 (99%) episodes of suspected sepsis. A clinically relevant finding in blood culture, bacteremia, was identified in 315/2,443 (13%) admissions from 309 unique patients. In 13/315 (4%) of blood cultures, there were multiple clinically relevant bacteria. The incidence of community onset bacteremia in adults was 203/100,000 (95% CI, 184–223). There was a more than 50-fold increase in the incidence of bacteremia, from 25 to 1,317 per 100,000, between the youngest and the oldest age groups. (Table 5). In those with Sepsis-3 sepsis at first admission, 283/1,299 (22%) had bacteremia and the median age was 78 years.

**Table 5:** Characteristics of 315 consecutive episodes of clinically relevant bacteremia in blood cultures among 2,462^1^ adult admissions for suspected community onset bacterial infection in Sweden.

N (%)	Total	Severe sepsis	Not severe sepsis
**Episodes**	2,462	482	1,980
**Bacteremia**	315 (13)	133 (28)	182 (9)
**Median age (years)**	78	80	77
**IQR**	67–85	69–86	65–83
**Men**	170 (54)	74 (56)	96 (53)
**Incidence/100,000**			
**All**	203	86	117
**Age groups**			
**18–49**	25	7	18
**50–64**	129	45	84
**65–74**	298	122	176
**75–84**	788	318	470
**85+**	1.317	708	609

^1^A blood culture was obtained before the start of antibiotic treatment in 2,443/2,462 (99%) of the patients. IQR, Interquartile range.

*Escherichia coli* was the most commonly identified bacterium, in 104 episodes (33%), followed by *Staphylococcus aureus* in 55 episodes (16%). Gram-negative bacteria were more prevalent (57%) than Gram-positives (47%). There were no methicillin-resistant *S*. *aureus* (MRSA), no vancomycin-resistant enterococci (VRE), and only two cases with extended spectrum beta-lactamase (ESBL)-producing *E*. *coli* in the blood cultures. (S5 Table).

### Septic shock and patients treated in the ICU

Of the 429 patients with severe sepsis at first admission, 64 also had septic shock according to Sepsis-2 criteria, giving an annual incidence of 41/100,000 (95% CI 33–51). Forty were men and 24 were women. The mean and median ages were 73 and 75 years, respectively. A bacterial diagnosis could be made in 35/64 (55%) of the cases, with the respiratory tract being the main focus of the infection in 39% of the cases. Forty-four out of 64 (67%) patients were treated in the intensive care unit (ICU).

Septic shock according to the new 2016 criteria could not be evaluated, since lactate analyses made in the ICU were analyzed on a local instrument and not entered into the electronic health record. However, 32 out of 64 patients with persisting hypotension when admitted to the ICU had a pre-ICU lactate of >2 mmol/L.

Altogether, 153/2,196 (7%) patients were treated in the ICU, 107/429 (25%) of the patients with severe sepsis, and 130/1,299 (10%) of the patients with Sepsis-3 sepsis.

### Focus of the infection

The respiratory tract was the most common focus of infection for both severe sepsis and Sepsis-3 sepsis (Table 6).

**Table 6:** The focus of infection in 2,072 consecutive patients with community onset bacterial infection. Values shown are percentages.

Focus of infection	Severe sepsis N = 429	Not severe sepsis N = 1,643	Sepsis-3 sepsis N = 1,299	Not Sepsis-3 sepsis N = 773
**Respiratory tract**	42	37	37	25
**Urinary tract**	19	7	18	21
**Abdomen**	12	16	12	14
**Skin and soft tissues**	6	14	6	21
**Bacteremia, focus unknown**	8	3	4	2
**Septicemia, unspecified**	5	0	2	0
**Gastrointestinal**	1	2	2	1
**Other specified infections**	2	14	13	10
**Infection, unspecified**	5	7	6	6

## Discussion

In this prospective chart-based population study in adults in southwestern Sweden we found an incidence of community onset severe sepsis of 276/100,000 (95% CI, 254–300). This incidence is high compared to previous studies using code-based abstraction [1]. However, the incidence of severe sepsis is lower in the present study than in three recent Scandinavian chart-based population studies [4–6].

Our retrospective analysis of the study population using Sepsis-3 criteria revealed an incidence of community onset Sepsis-3 sepsis of 838/100,000 (95% CI, 798–877). To our knowledge, there are no other chart-based studies of the performance of the SOFA criteria for sepsis as applied to a whole population in a real-life setting. Our incidence is higher than that in the Mellhammar *et al*. [6] study of all types of sepsis, including hospital acquired infections.

The different results obtained in these Scandinavian studies can be explained by the differences in methodology, populations studied, criteria used, and by how infections were defined.

Advanced age is a well-known risk factor for sepsis. In this study, the incidence in sepsis increased more than forty-fold between the youngest and the oldest age groups by either definition. The median age was high, 78 years, both in the group having severe sepsis, as well as in the group having Sepsis-3 sepsis. This is in accordance with the study of Mellhammar *et al*.[6], who reported median ages of 78 and 80 years for severe sepsis and Sepsis-3 sepsis, respectively.

The incidence of severe sepsis was higher in men than in women. This was shown already by Angus *et al*. in 2001 [17] and has been verified by other groups [5, 6]. In our population, the incidence of severe sepsis in men increased from the age-groups of 65–74 and older, being 70% higher among those who were ≥85 years of age. To our knowledge, this has not been described previously.

Applying the Sepsis-3 criteria to this study cohort increased the proportion of patients having organ dysfunction almost 3-fold, from 20% using severe sepsis criteria to 59% using Sepsis-3 criteria. Yet, the median age in both groups remained the same, 78 years. The main reason for the higher incidence of Sepsis-3 was the difference in the respiratory criterion for organ dysfunction. As many as 78% of the patients with Sepsis-3 sepsis were diagnosed with sepsis due to the respiratory criterion only, which is in accordance with the values of 80% reported by Mellhammar *et al*.[6]. This is not surprising, since the criterion for respiratory dysfunction, ≥2 points in the SOFA score, is equivalent to an oxygen saturation of <92% on room air breathing, irrespective of whether pneumonia is present or not.

Examinations of the normal levels of oxygen saturation show decreases with increasing age, especially in persons who are >70 years of age [18–20], and also decreases when the measurement is made with the patient in the supine position [21]. To avoid over-diagnosing patients as having sepsis, the respiratory criterion for organ dysfunction should be stricter.

This difference in incidences between Sepsis-2 and Sepsis-3 sepsis in a general population stands in sharp contrast to the situation in an ICU setting. Shankar Hari *et al*. [22] found a very small difference between patients in the ICU diagnosed with organ dysfunction, regardless of whether Sepsis-2 or Sepsis-3 criteria were used. Our findings illustrate the difference in outcome between the Sepsis-2 and Sepsis-3 criteria when applied to a group of patients that is less sick, and often of high age, compared to those treated in the ICU.

Hypoperfusion and hypotension were the most commonly identified organ dysfunctions according to the severe sepsis criteria and appeared at constant rates across all the age groups. Respiratory and cerebral dysfunctions increased with age. Hepatic dysfunction was rare, and the liver was never the only dysfunctional organ. Cerebral dysfunction was uncommon in patients aged <50 years. This is in accordance with the clinical observation that hypotension and high lactate are more common in younger patients with severe sepsis than is acute change of mental status.

Hypoperfusion, defined as a lactate level >1 mmol/L above the upper reference limit, was identified in just over half of the patients with severe sepsis. This is higher than reported in most other studies, even two large studies on septic shock [23, 24], in which about 20% of the patients had elevated lactate levels. The main reason is most likely that we used venous lactate. For physiologic reasons, venous lactate levels are normally 0.5–1.0 mmol/L higher than those of arterial lactate [25]. This is seen also in patients with sepsis [26, 27]. Though not part of the Sepsis-3 criteria, Rhee *et al*., also found increased levels of lactate in 52% of almost 174,000 patients with Sepsis-3 sepsis [3].

Bacteremia was identified in 13% of all the admissions for suspected community onset sepsis, similar to previously reported in population-based studies [4–6]. This corresponds to an annual incidence in adults of 203/100,000, which is higher than most previous studies. One Swedish study by Holmbom *et al*. [28], which also included children, found an incidence for the same time period of community onset bloodstream infections (BSI) of 157/100,000. A contemporary Norwegian study carried out by Mehl *et al*. [29], found an incidence of 223/100,000, including hospital-acquired BSI. A similar Danish study from 2008 by Nielsen *et al*. [30] found an incidence of all BSI of 199/100,000. In historical studies, often including HAIs, the incidence has varied in the range of 80–189/100,000, as described by Laupland *et al*. [31]. The reason for the high incidence in our study is the very high rate (>99%) of blood cultures drawn before the start of intravenous antibiotic treatment.

A strength of the present study is that it was prospective and was performed in a public healthcare system that is free of charge to the patients and with open access. Logistic factors of the present study, and the high level of compliance to the drawing blood cultures before the start of intravenous antibiotic treatment, make us to believe that we did find the majority of the patients that we intended to find.

The study has weaknesses. First, it is a single-center study with a relatively low number of patients. Second, it was performed over a period of 9 months, whereas the data were extrapolated to a period of 12 months. Third, the patients in general wards were not monitored as closely as the patients in the ICU, so adequate documentation and sampling at the time-points when the patients were the most sick could be missed. Fourth, we missed to include 7–10% of the patients we intended to include and 10% of the protocols were never returned, which is not an insignificant amount. Therefore, the incidences should probably be even higher.

## Conclusions

This is one of the few chart-based prospective studies conducted on the incidences of sepsis in adults within a whole population. The incidence of 276/100,000 observed for community onset severe sepsis is lower than the incidences reported in recent Scandinavian studies. The incidence of community onset Sepsis-3 sepsis, 838/100,000, is the highest reported to date. The difference between the incidences of Sepsis-2 and Sepsis-3 sepsis is mainly due to the less-stringent criterion used for respiratory dysfunction in Sepsis-3 sepsis. The incidence of community onset bacteremia, 203/100,000, was high compared to other studies, probably due to the high rate of blood cultures.

The median age of patients with severe sepsis, Sepsis-3 sepsis and with bacteremia was high, 78 years. The incidence of each of those increased, as was already known, with increasing age, although the magnitude of the increase between the youngest and the oldest age groups was considerable, >40-fold.

## Supporting information

S1 Text Definitions of infections. (PDF)

S1 Table Population categorized according to age group and gender. Incidences of severe sepsis and Sepsis-3.
(PDF)

S2 Table Swedish criteria (2011) for hypotension, hypoperfusion, and organ dysfunction in severe sepsis and septic shock in adults. (PDF)

S3 Table SOFA scores. (PDF)

S4 Table Definitions of comorbidities. (PDF)

S5 Table Bacterial findings in 315 blood cultures. (PDF)
