# Supplementary material for: Incidences of community onset severe sepsis, Sepsis-3 sepsis, and bacteremia in Sweden – A prospective population-based study
Source: PLoS One. 2019 Dec 5;14(12):e0225700. doi: 10.1371/journal.pone.0225700 (PMC6894792; doi:10.1371/journal.pone.0225700)
Supplement: S1 Text — (PDF) [file pone.0225700.s001.pdf]

S 1 Text. Definitions of infections.

For infection diagnosis, the definitions set by the Swedish Infectious Disease Society were used when available. Apart from pneumonia, the guidelines are in Swedish and published online only on the Society homepage: www.infektion.net/vardprogram. There are guidelines for urinary tract infections, endocarditis, bone and joint infections, bacterial CNS infections, viral CNS infections, and *Clostridium difficile* infections. For other diagnoses, the clinician's discharge diagnosis was used unless microbiologic or imaging findings prompted correction by any of the reviewers. The definitions used for the most common infections, pneumonia, and urinary tract infections are:

Pneumonia

Pneumonia is defined as "an infection of the lung parenchyma with symptoms and clinical findings compatible with acute lower respiratory tract infection also having radiological evidence of pneumonia" [16].

Urinary tract infection

A febrile urinary tract infection is defined as "an infection emanating from the urinary tract with fever and general symptoms, with or without focal urinary tract symptoms". Pyelonephritis is defined as "an infection localized to the renal parenchyma and collecting duct and characterized by fever, general symptoms, flank pain, and palpation tenderness over the kidney". In men, the microbiologic criterion is ≥103 colony forming units (CFU)/mL for a known primary or secondary urinary tract pathogen. In women, it is ≥103 CFU/mL for a primary pathogen and ≥104 CFU/mL for a secondary pathogen.

Viral respiratory tract infections

A viral respiratory tract infection was diagnosed during the winter season using a laboratory-developed PCR for Influenza A, Influenza B, and Human orthopneumovirus (formerly known as Human respiratory syncytial virus), used at the discretion of the treating clinician. During 10 weeks of the Winter season, we also used two different Multiplex PCRs for respiratory pathogens as part of the sepsis study, as previously described [14]. The results by either method were used in the evaluation of infection etiology, provided there were signs of an acute respiratory illness.
