# Supplementary material for: Incidences of community onset severe sepsis, Sepsis-3 sepsis, and bacteremia in Sweden – A prospective population-based study
Source: PLoS One. 2019 Dec 5;14(12):e0225700. doi: 10.1371/journal.pone.0225700 (PMC6894792; doi:10.1371/journal.pone.0225700)
Supplement: S1 Table — Incidences of severe sepsis and Sepsis-3. (PDF) [file pone.0225700.s002.pdf]

S 1 Table. Population characteristics and incidences of severe sepsis and Sepsis-3 sepsis.

Age group (years)	Population (N)	Male: Female ratio	Severe sepsis (N)	Incidence/ 100,000/ year	Sepsis-3 sepsis (N)	Incidence/ 100,000/ year
All ≥18	206,900	1.00	429	276	1,299	838
Men	103,300		231	298	708	913
Women	103,600		198	255	591	763
18–49	100,900	1.08	29	38	96	127
Men	52,300		17	43	57	145
Women	48,600		12	33	39	107
50–64	50,800	1.03	53	139	176	462
Men	25,700		25	130	98	508
Women	25,000		28	149	78	416
65–74	29,500	1.00	99	447	273	1,230
Men	14,600		58	530	159	1,450
Women	14,900		41	367	114	1,020
75–84	17,600	0.80	127	962	399	3,020
Men	7,800		71	1,213	227	3,880
Women	9,800		56	761	172	2,340
≥85	8,100	0.55	121	1,991	355	5,840
Men	2,900		60	2,758	167	7,680
Women	5,200		61	1,565	188	4,820
