# Supplementary material for: Incidences of community onset severe sepsis, Sepsis-3 sepsis, and bacteremia in Sweden – A prospective population-based study
Source: PLoS One. 2019 Dec 5;14(12):e0225700. doi: 10.1371/journal.pone.0225700 (PMC6894792; doi:10.1371/journal.pone.0225700)
Supplement: S2 Table — (PDF) [file pone.0225700.s003.pdf]

S 2 Table. The Swedish 2011 consensus definition and criteria for severe sepsis and septic shock. The criteria presuppose that changes have occurred from fairly normal organ function and are not expected to have causes other than the systemic inflammatory reaction [11].

**Sepsis**	Suspected infection *plus* ≥2 SIRS[1]-criteria
**Severe sepsis**	Sepsis *or* documented infection *plus* either hypotension, hypoperfusion *or* organ dysfunction
Hypotension	Systolic blood pressure ≤90 mmHg *or* mean arterial pressure ≤70 mmHg
Hypoperfusion	Blood lactate >3 mmol/L or >1 mmol/L above the upper reference limit, *or*, base excess ≤-5 mmol/L
Organ dysfunction:	
Respiratory	PaO_2/FiO_2 <33 kPa (corresponding to 86% oxygen saturation on air breathing) *or* PaO_2/FiO_2 <27 kPa (corresponding to 78% oxygen saturation on air breathing) if the lung is the focus of infection.
Renal	<0.5 mL urine/kg/2 hours despite adequate volume resuscitation
Hematologic	Thrombocytes <100 $\times 10^6$/mL, *or* INR >1.5 or APTT >60 seconds
Cerebral	Acute change of mental status
Hepatic	Serum bilirubin >45 µmol/L
**Septic shock**	Persisting hypotension despite adequate volume resuscitation (500–1,000 mL of crystalloid given within 30 minutes) *plus* either hypoperfusion or organ dysfunction

[1]SIRS criteria consist of: a) heart rate >90 beats/min; b) Respiratory rate >20 breaths/min; c) body temperature >38.0°C or <36.0°C; d) Leukocyte count >12.0 × 10^9/mL or <4.0 × 10^9/mL or >10% bands.
PaO_2, Partial pressure of oxygen in arterial blood; FiO_2, fraction of oxygen in the inhaled air; INR, International normalized ratio; APTT, activated partial thromboplastin time.

Septic shock is defined as severe sepsis with hypotension not responding to adequate fluid treatment (500–1,000 mL crystalloid fluid given in 30 minutes).
