# Supplementary material for: Incidences of community onset severe sepsis, Sepsis-3 sepsis, and bacteremia in Sweden – A prospective population-based study
Source: PLoS One. 2019 Dec 5;14(12):e0225700. doi: 10.1371/journal.pone.0225700 (PMC6894792; doi:10.1371/journal.pone.0225700)
Supplement: S3 Table — (PDF) [file pone.0225700.s004.pdf]

**S 3 Table. SOFA scores.** Modified after Mellhammar, *et.al.* [6].

| **Variable** | **SOFA-score** 0 | 1 | 2 | 3 | 4 |
|---|---|---|---|---|---|
| Respiration: $PaO_2/FiO_2$, kPa | >53 | ≤53 | ≤40 | ≤27 | ≤13 |
| Corresponding $SpO_2$ % without supplementary $O_2$* | ≥96 | <96 | <92 | <79 | <49 |
| Coagulation: Thrombocytes, x $10^9$/l | >150 | ≤150 | ≤100 | ≤50 | ≤20 |
| Liver: Bilirubin, µmol/l | <20 | 20-32 | 33-101 | 102-204 | >204 |
| Hypotension: mean arterial pressure, MAP mmHg | ≥70 | ≥70 | Dopamine ≤5[1]<br>Dobutamine[2] | Dopamine >5[1]<br>Adrenaline ≤0.1[1]<br>Noradrenaline ≤0.1[1] | Dopamine >15[1]<br>Adrenaline >0.1[1]<br>Noradrenaline >0.1[1]<br>Levosimendan[2]<br>Vasopressin[2] |
| Cerebral: GCS | 15 | 13-14 | 10-12 | 6-9 | 3-5 |
| RLS* | 1 | 2 | 3 | 4-5 | 6-8 |
| Renal: Creatinine, µmol/l | <110 | 110-170 | 171-299 | 300-400 | >440 |
| Diuresis, ml/day | ≥500 | ≥500 | ≥500 | <500 | <200 |

1) Catecholamine doses are given as µg/kg/min.
2) Regardless of dose.
$FiO_2$, Fraction of inspired oxygen; $PaO_2$, partial pressure of oxygen; $SaO_2$, arterial oxygen saturations; GCS, Glasgow Coma Scale; RLS, Reaction Level Scale.
*Not part of the original SOFA score

Instead of the Glasgow Coma Scale (GCS), the Reaction Level Scale (RLS) [Starhammar *et.al.* 1982] was used for acute changes in mental status, where RLS 2 corresponds to GCS 13–14, RLS 3 to GCS 10–12, RLS 4–5 to GCS 7–9, and RLS 6–8 to GCS <3–5. For respiratory dysfunction, the oxygen saturation values in the arterial blood gas were used, when available. Otherwise, oxygen saturation was estimated using the values acquired by pulse oximetry. The oxygen saturation values were corrected for oxygen supplementation according to data from the Swedish Intensive Care Register, where 1, 2, 3 and 4 Liters of oxygen delivered by nasal catheter or 5 and 10 Liters delivered using an oxygen mask are estimated to yield inhaled oxygen fractions, $FiO_2$, of 0.22, 0.24, 0.26, 0.28, 0.35 and 0.6, respectively, and using the formula described by Severinghaus [Severinghaus 1979].
