# Supplementary material for: Incidences of community onset severe sepsis, Sepsis-3 sepsis, and bacteremia in Sweden – A prospective population-based study
Source: PLoS One. 2019 Dec 5;14(12):e0225700. doi: 10.1371/journal.pone.0225700 (PMC6894792; doi:10.1371/journal.pone.0225700)
Supplement: S4 Table — (PDF) [file pone.0225700.s005.pdf]

S 4 Table. Definitions of comorbidities.

Comorbidities were divided into eight categories, all of which had to have been previously diagnosed:

1.	Chronic cardiovascular disease	any type of chronic cardiovascular disease, such as hypertension, angina pectoris, previous myocardial infarction, previous stroke, or previous surgery due to arterial insufficiency
2.	Chronic respiratory disease	any type of chronic respiratory disease, such as chronic obstructive pulmonary disease, chronic asthma or pulmonary fibrosis
3.	Chronic renal disease	any renal disease leading to impaired renal function
4.	Chronic liver disease	any liver disease leading to impaired liver function
5.	Diabetes mellitus	a previous diagnosis of diabetes mellitus
6.	Malignancy	any ongoing malignancy or a malignancy within the past 5 years
7.	Immunosuppression	either a primary or acquired immunodeficiency, cortisone treatment equivalent to >15 mg of prednisolone daily for >3 weeks, or any other chemotherapy
8.	Other comorbidities	any other significant chronic disease, e.g., Alzheimer's disease or multiple sclerosis
