# Supplementary material for: Incidences of community onset severe sepsis, Sepsis-3 sepsis, and bacteremia in Sweden – A prospective population-based study
Source: PLoS One. 2019 Dec 5;14(12):e0225700. doi: 10.1371/journal.pone.0225700 (PMC6894792; doi:10.1371/journal.pone.0225700)
Supplement: S5 Table — (PDF) [file pone.0225700.s006.pdf]

S5 Table. Bacterial findings in 315 blood cultures in Skaraborg sepsis study during 9 months 2011-2012.

ICD-10 code	Species	n
**B950**	*Streptococcus pyogenes*	10
**B951**	*Streptococcus agalactiae*	5
**B952**	*Enterococcus faecalis/faecium*	11
**B953**	*Streptococcus pneumoniae*	21
**B954**	*Streptococcus species*	20
	S. dysgalactiae (Group C or G)	*(6)*
	S. anginosus	*(3)*
	S. intermedius	*(2)*
	S. constellatus	*(1)*
	S. mitis	*(4)*
	S. gallolyticus	*(1)*
	S. gordonii	*(1)*
	Aerococcus urinae	*(2)*
**B956**	*Staphylococcus aureus*	55
**B957**	*Coagulase negative staphylococcus*	5
**B961**	*Klebsiella pneumoniae*	24
**B962**	*Escherichia coli*	104
**B963**	*Haemophilus influenzae*	5
**B964**	*Proteus mirabilis/morgani*	11
**B965**	*Pseudomonas aeruginosa*	9
**B966**	*Bacteroides fragilis*	5
**B967**	*Clostridium perfringens*	1
**B968**	Other specified bacteria	29
	Klebsiella oxytoca	*(4)*
	Enterobacter claoacae	*(2)*
	Citrobacter koseri	*(1)*
	Citrobacter freundii	*(1)*
	Serratia marscesens	*(2)*
	Acinetobacter	*(2)*
	Bacillus cereus	*(1)*
	Raoultella ornithinolytica	*(2)*
	Pasteurella multocida	*(1)*
	Parvimonas micra	*(1)*
	Eikinella corrodens	*(1)*
	Alcaligenes xylosoxidans	*(1)*
	Rothia mucilanginos	*(1)*
	Moraxella catarrhalis	*(1)*
	Neisseria elongata	*(1)*
	Actinobaculum schaalii	*(1)*
	Cutibacterium acnes	*(1)*
	Bacteroides uniformis	*(2)*
	Bacteroides thetaiotamicron	*(2)*
	Clostridium septicum	*(1)*
